# Surface Hardness Impairment of Quorum Sensing and Swarming for *Pseudomonas aeruginosa*

**DOI:** 10.1371/journal.pone.0020888

**Published:** 2011-06-07

**Authors:** Nachiket G. Kamatkar, Joshua D. Shrout

**Affiliations:** 1 Department of Civil Engineering and Geological Sciences, University of Notre Dame, Notre Dame, Indiana, United States of America; 2 Department of Biological Sciences, University of Notre Dame, Notre Dame, Indiana, United States of America; 3 Eck Institute for Global Health, University of Notre Dame, Notre Dame, Indiana, United States of America; Vrije Universiteit Brussel, Belgium

## Abstract

The importance of rhamnolipid to swarming of the bacterium *Pseudomonas aeruginosa* is well established. It is frequently, but not exclusively, observed that *P. aeruginosa* swarms in tendril patterns—formation of these tendrils requires rhamnolipid. We were interested to explain the impact of surface changes on *P. aeruginosa* swarm tendril development. Here we report that *P. aeruginosa* quorum sensing and rhamnolipid production is impaired when growing on harder semi-solid surfaces. *P. aeruginosa* wild-type swarms showed huge variation in tendril formation with small deviations to the “standard” swarm agar concentration of 0.5%. These macroscopic differences correlated with microscopic investigation of cells close to the advancing swarm edge using fluorescent gene reporters. Tendril swarms showed significant *rhlA-gfp* reporter expression right up to the advancing edge of swarming cells while swarms without tendrils (grown on harder agar) showed no *rhlA-gfp* reporter expression near the advancing edge. This difference in rhamnolipid gene expression can be explained by the necessity of quorum sensing for rhamnolipid production. We provide evidence that harder surfaces seem to limit induction of quorum sensing genes near the advancing swarm edge and these localized effects were sufficient to explain the lack of tendril formation on hard agar. We were unable to artificially stimulate rhamnolipid tendril formation with added acyl-homoserine lactone signals or increasing the carbon nutrients. This suggests that quorum sensing on surfaces is controlled in a manner that is not solely population dependent.

## Introduction

*Pseudomonas aeruginosa* is a bacterium that survives in many diverse environments. Well known as both a clinically and environmentally relevant organism, much of *P. aeruginosa* growth in these different niches is perceived to be as surface-associated biofilms [1]. The surface characteristics, not just the nutrient conditions, for these diverse growth environments are highly variable. We were interested to understand how *P. aeruginosa* freely colonizes such different surface environments.

*P. aeruginosa* and many other bacteria, including species found in diverse soil and water environments such as *Serratia liquefaciens*, *Vibrio cholerae*, *Vibrio parahaemolyticus*, *Proteus mirabilis*, and *Bacillus subtilis*, colonize surfaces by swarming [2], [3], [4], [5]. Swarming is typically studied in the laboratory using semisolid (e.g., agar) plate assays. Swarming bacteria spread over surfaces by flagellar propulsion within a thin-liquid film layer on top of these agar plates [2], [3], [4], [6], [7]. For most swarming bacteria, the spreading properties of this high-population liquid swarm layer are enhanced by production of a surfactant by the bacteria [3]. Swarming can also occur without a surfactant; *Salmonella enterica*, for example, swarms with the aid of an osmotic agent that is not a surfactant [8].

*P. aeruginosa* swarming is aided by its production of the glycolipid surfactant di-rhamnose-β-hydroxyalkanoyl-β-hydroxyalkanoate (rhamnolipid) [9], [10], [11]. Expression of the rhamnolipid biosynthetic operon is initiated in a population-dependent manner described as quorum sensing [12]. *P. aeruginosa* utilizes two acyl-homoserine (AHL) signals to regulate different sub-sets of genes. These two AHLs have specific affinity to two LuxR-homolog transcriptional regulators, LasR and RhlR; this regulation occurs in series where a fully-induced Las-system initiates activation of the Rhl-system. Quorum sensing controls rhamnolipid production through the Rhl-system (named for rhamnolipid) where the RhlR activates expression of the *rhlA* and *rhlB* genes only when sufficient butyryl homoserine lactone is present [13], [14]. RhlA converts β-hydroxydecanoyl-ACP to halo-alkanoic acid (HAA) [10], [15] and RhlB is a rhamnosyltransferase that converts HAA to mono-rhamnolipid [10], [13]. (The final rhamnolipid synthesis gene, *rhlC*, encodes for a rhamnosyltransferase that converts mono-rhamnolipid to di-rhamnolipid [16]. The *rhlC* gene is located elsewhere on the chromosome and is not regulated by RhlR.) These di-rhamnolipid precursors act similarly to di-rhamnolipid in aiding swarming; HAAs, mono-rhamnolipid, and di-rhamnolipid all act as surfactants to lower surface tension [10]. Tremblay et al. [17], however, have shown that these molecules have distinctive chemotactic and diffusive properties. Di-rhamnolipid acts as an attractant while the precursor HAAs can act as repellants to *P. aeruginosa*. There also appear to be growth conditions where rhamnolipid is not required, but still aids, *P. aeruginosa* swarming [18], [19]. Therefore, while rhamnolipid synthesis is well-described and the ability to improve surface spreading of *P. aeruginosa* is known, the spatial and temporal actions of rhamnolipid and its precursors on surfaces are less defined.

Most images of *P. aeruginosa* swarms show cell spreading in fractal or tendril patterns; development of these tendrils requires rhamnolipid [9], [17], [20], [21], [22]. However, others have shown that *P. aeruginosa* also swarms as an expanding circle [10], [11], [18], [19], [23], [24]. Differences in these varied reports for *P. aeruginosa* swarming seem to be explained by a variety of factors including strain effects, media composition, and surface hardness.

We became interested in the influence of surface hardness upon swarming and tendril formation. Higher agar or “hard” surfaces are known to limit swarming [11], [25] as do overdried plates [26]. We found that varying the concentration of the agar within a small range greatly altered swarming patterns and overall spreading. We hypothesized that surface properties affect rhamnolipid production, which subsequently affects swarming. We provide evidence that hard agar surfaces limit the initiation of quorum sensing and rhamnolipid production in very close proximity to the advancing edge of swarming cells, which is sufficient to dominate the resultant swarm phenotype. Adding exogenous AHL signals or increasing substrate carbon did not alter wild-type swarming. Because we were unable to artificially stimulate tendril formation on hard agar, this suggests that quorum sensing on surfaces is not solely population dependent.

## Results

### Rhamnolipid mediated swarming varied with surface conditions

**Figure 1:**
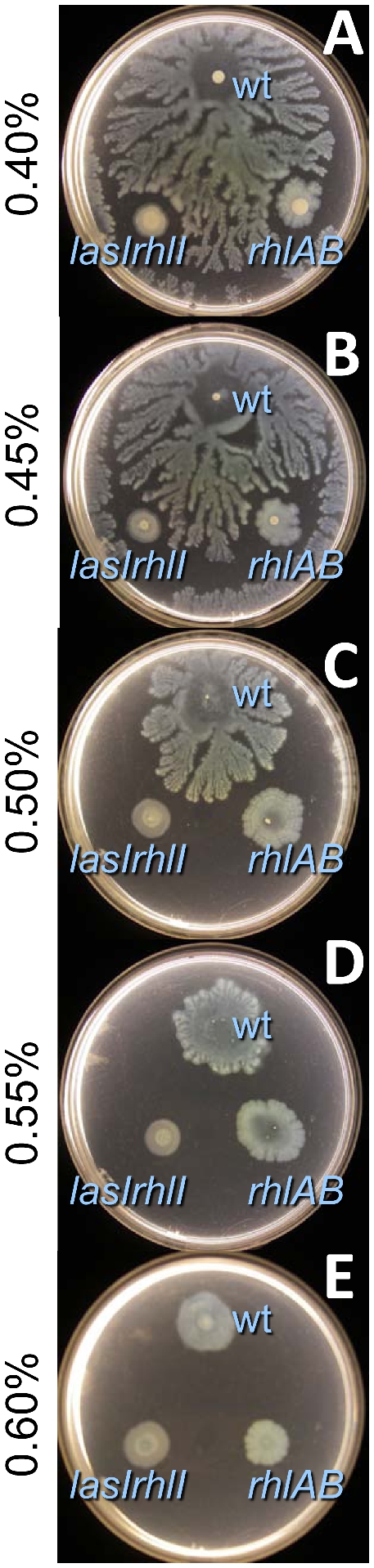
*P. aeruginosa* swarming for wild-type, *lasIrhlI*-mutant, and *rhlAB*-mutant on different % noble agar with FAB-glutamate medium. Plate assays were incubated at 30°C for 60 hours.

For *P. aeruginosa*, the transition between robust swarming on “soft agar” and low motility on “hard agar” can be observed with examination of plate assays containing a range of 0.4%–0.6% Noble agar (Figure 1). Small decreases in agar from 0.5% (the “standard” swarm agar for *P. aeruginosa*) yield very apparent increases in swarm diameter and increases in fractal heterogeneity (i.e., tendrils) of the swarm zone that have been observed in several previous studies [9], [11], [20]. (We subsequently designate 0.40%–0.45% agar as “soft” and 0.6% agar as “hard”.) As expected, neither a rhamnolipid-deficient strain (*rhlAB*) nor a quorum sensing signal mutant (*lasIrhlI*) yielded tendrils at any agar concentration investigated. While swarming of the wild-type and each of these mutants was greatly reduced on hard agar, the overall spreading was slightly greater than that of a completely impaired rhamnolipid-flagellar double mutant (Figure S1). Thus, there appeared to be at least two motility mechanisms that contribute to swarming observed for these plate assays: production of rhamnolipid and flagellar activity. We further investigated surface variation of rhamnolipid-dependent swarming.

### Rhamnolipid gene expression (rhlA) differed greatly at the swarm edge

Since swarm tendrils are due to rhamnolipid, we questioned if a lack of tendrils was due to insufficient rhamnolipid production. Adapting the methylene blue rhamnolipid plate assay [27] utilized in several studies, we observed that wild-type qualitatively produces similar rhamnolipid amounts on both soft and hard agar (Figure S2). The zones of clearing that indicated production of surfactant were not differentiable between agar types. Hard agar did not prevent rhamnolipid production. These methylene blue indicator plates did not, however, provide insight into actual rhamnolipid production levels during swarming; the CTAB component of these plate assays is toxic to bacteria [28] and swarming of *P. aeruginosa* was impaired on these plates. While the potential to produce rhamnolipid may be equal on these two agar surfaces, this particular assay provides little information of how *P. aeruginosa* behaves under more optimal conditions.

**Figure 2:**
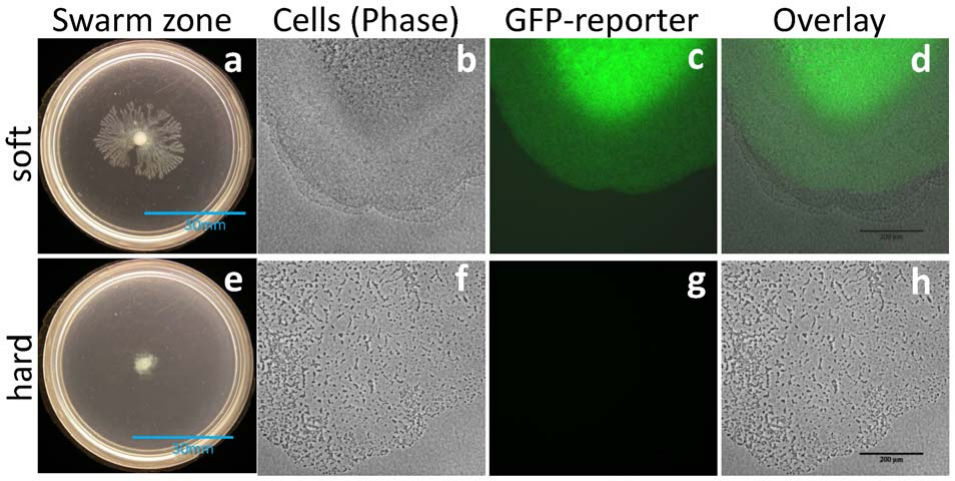
*P. aeruginosa* swarming after 40 hours for a rhamnolipid fluorescence reporter wild-type strain growing on soft (0.4%) and hard (0.6%) agar (a,e) entire swarm colony; (b,f) phase-contrast image of swarm edge; (c,g) fluorescence of P*_rhlA_*::*gfp* fusion; (d,h) overlay of b+c and f+g showing proximity of rhamnolipid production to the swarm edge. Whole plate scale bar = 30 mm; overlay scale bar = 200 µm.

We then examined expression of a rhamnolipid gene fluorescent reporter (as in [29]) to gauge potential differences in rhamnolipid synthesis *in situ* during swarming. The reporter construct utilizes the promoter region of *rhlA* (required to make HAA rhamnolipid precursors) fused to green fluorescent protein. We observed that *P. aeruginosa* induction of *P_rhlA_::gfp* is greater at the advancing edge of soft agar swarms compared to those grown on hard agar (Figure 2). After 40 hours, the formation of swarm tendrils corresponded to fluorescence of P*_rhlA_::gfp* in very close proximity (≤20 µm) to the advancing edge of swarming bacteria on soft agar (Figure 2d). On hard agar, however, when no tendrils formed, the fluorescence detected near the swarm edge was barely above background (Figure 2g) and 37× less than the expression observed near the swarm edge on soft agar (Figure 2c). These *rhlA* expression differences were observed only at the swarm edge; fluorescence toward the center of swarms (>800 µm from edge) was bright and indistinguishable on all agar types (Figure S3). The overall bacterial population showed no evidence of limited rhamnolipid production—most of the hard agar-grown colony area is bright with P*_rhlA_::gfp* and produced rhamnolipid on methylene blue indicator plates. This suggests that the ability to form swarm tendrils depends highly upon localized rhamnolipid production close to the advancing edge of swarming cells.

Localized effects were also observed when examining the cell density patterns at the advancing swarm edge. The phenotypes of cell distribution at the advancing edge on soft and hard agar were quite different after 40 hours. The phase image of the advancing swarm growing on soft agar showed a continuous swarm of bacterial cells (Figure 2b). After 40 hours on hard agar, however, the swarm was not continuous but rather an assemblage of several distinct aggregates; there was no defined edge of the swarm zone (Figure 2f). While the cell distribution patterns between agar types were dissimilar, cells were motile on each of these surfaces. Many motile cells were observed on either soft or hard agar in real-time (15 fps) within these slowly advancing swarms (Movies S1 and S2).

The localized P*_rhlA_*::*gfp* fluorescence and cell distribution (phase image) corresponding with tendril formation was evident after many hours of growth. Tendril swarms on soft agar show increased and earlier expression of P*_rhlA_*::*gfp* fluorescence. Differentiation of swarm phenotypes between agar conditions manifested after roughly one day (a time course is shown as Figure S4). Early on (after 12 hours), when swarms were the same by eye, cells had spread ≤5 mm and cell density was identical by magnification for cells grown on both agar types; the P*_rhlA_*::*gfp* fluorescence was also indistinguishable for these 12 hour swarms. At 27 hours, the overall swarm pattern showed slight differences that were more apparent with magnification; at higher agar concentrations, some cells accumulated into dense aggregates (c.f. Figure S4c and S4d). The differences in *P_rhlA_::gfp* fluorescence were more substantial at this time point as soft agar swarms showed greater and more confluent fluorescence than for cells growing on hard agar. Table 1 lists the relative intensity of the *P_rhlA_::gfp* fluorescence reporter at the swarm edge on these different surfaces over time. By 40 hours (Figure 2) and 63 hours (Figure S4), both the *P_rhlA_::gfp* expression and cell density patterns were very different on soft versus hard agar. Tendril swarms on soft agar show edge populations with a dense continuous edge of swarming bacteria expressing much greater *P_rhlA_::gfp* fluorescence. As stated above for the 40 hour images, inspection of cells away from the swarm edge towards the center showed bright fluorescence and dense continuous bacterial communities (>800 µm away from swarm edge) at these later time points (Figure S3).

**Table 1:** Fluorescence over time for P*_rhlA_::gfp* reporter of cells at swarm edge*.

Time	soft (0.4%) agar	hard (0.6%) agar
12	0.1	0.2
27	100	80
40	98	2.7
63	100	<0.05

*relative% of maximum intensity for all measurements.

### Quorum sensing expression differed between hard and soft agar

**Figure 3:**
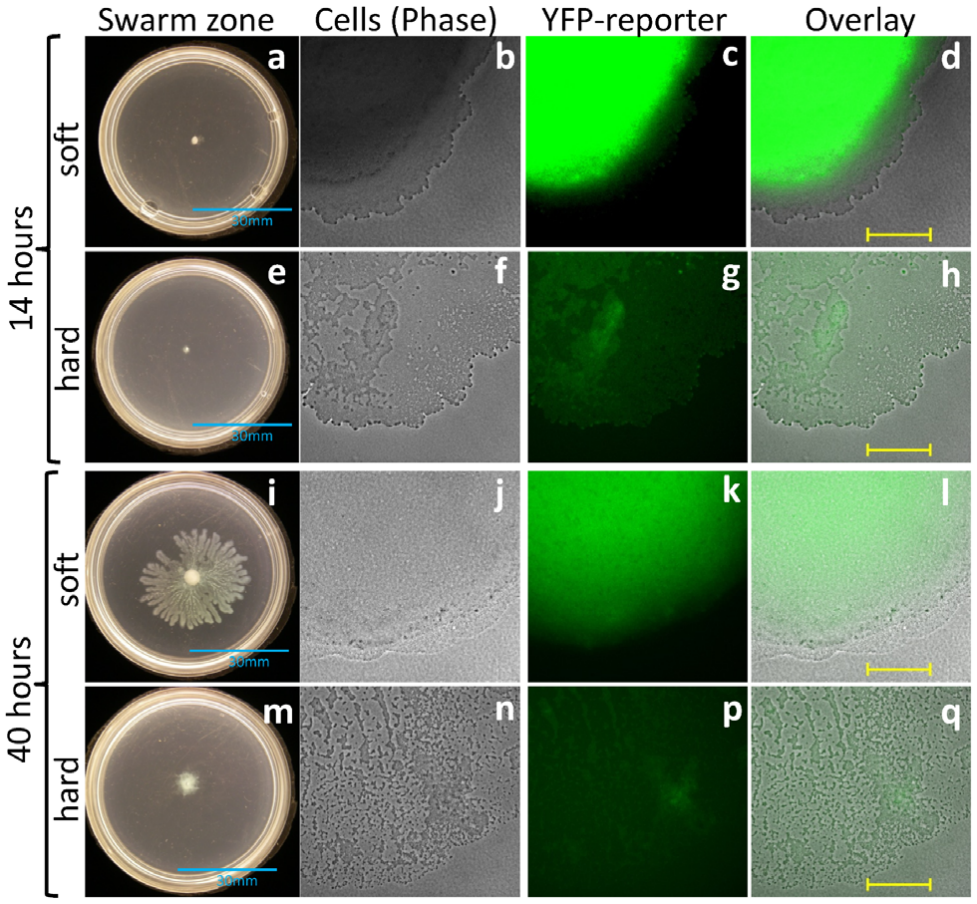
*P. aeruginosa* swarming after 14 and 40 hours for an early quorum sensing fluorescence reporter wild-type strain growing on soft (0.4%) and hard (0.6%) agar (a,e,i,m) entire swarm colony; (b,f,j,n) phase-contrast image of swarm edge; (c,g,k,p) fluorescence of P*_rsaL_*::*yfp*(ASV) fusion; (d,h,l,q) overlay of b+c, f+g, j+k and n+p showing proximity of initiated quorum sensing (*rsaL*)to the swarm edge. Whole plate scale bar = 30 mm; overlay scale bar = 200 µm.

Because synthesis of rhamnolipid requires a functioning *P. aeruginosa* quorum sensing cascade, we investigated if differences in quorum sensing could explain these localized differences in rhamnolipid and tendril formation. To assess onset of quorum sensing during swarming, we examined a fluorescence reporter of the gene *rsaL*. The gene *rsaL* is documented as among the earliest and most robust genes regulated by quorum sensing in *P. aeruginosa* [30], [31], [32]; RsaL appears to act as a global regulator that acts to provide homeostasis during quorum sensing to prevent accumulation of AHLs [33]. We used a highly sensitive P*_rsaL_*::*yfp*(ASV) fusion to assess those bacterial populations exhibiting a minimum quorum sensing response. Prior to tendril formation after 14 hours growth (when soft and hard agar swarms have not differentiated), fluorescence intensity of the *rsaL* reporter is much greater for cells growing on soft agar (11.5× greater pixel intensity than hard agar) over the same area (Figure 3c, g). This was despite similar distributions of cells on these surfaces at this early time point (Figure 3b, f). The population near the edge grown on hard agar never showed equal *rsaL* fluorescence to that achieved on soft agar. After 40 hours, the *rsaL* reporter showed 3.4× greater fluorescence on soft agar at 40 hours compared to hard agar (Figure 3k, p). Therefore, we explain the limited expression of *rhlA* (discussed above) by a more general limitation of quorum sensing activity on hard agar. As with the *rhlA* reporter images, fluorescence of P*_rsaL_*::*yfp* was bright and indistinguishable toward the center of all swarms (not shown); differences in *rsaL* fluorescence were only apparent at the advancing edge of these swarm populations.

**Figure 4:**
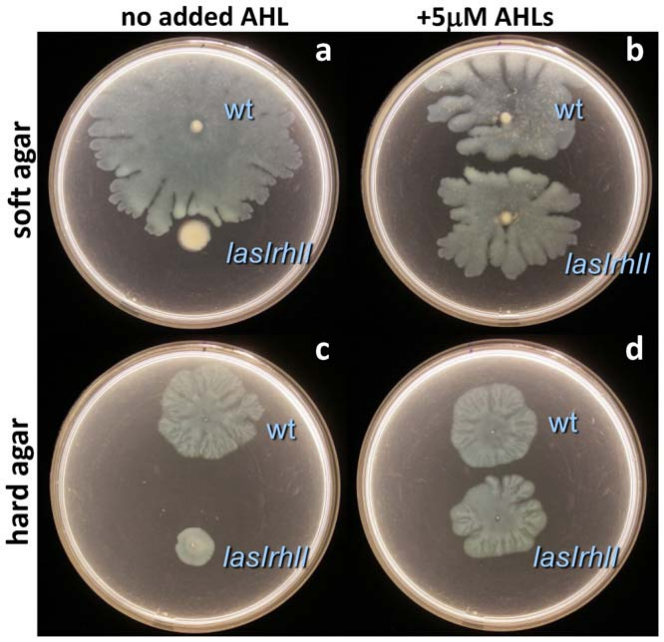
Swarming of wild type and *lasIrhlI*-mutant growing on FAB-glutamate plate assays amended with 3-oxo-dodecanoyl homoserine lactone and butyryl homoserine lactone (5 µM each) on soft (0.4%) and hard (0.6%) agar. Plates were incubated for 60 hours.

These differences in *rsaL* reporter fluorescence during growth on these two agar surfaces suggested that quorum sensing was not fully induced for the bacterial population at the swarm edge on harder agar surfaces. This was despite an apparently large population of bacteria that might otherwise be predicted to have been induced for quorum sensing. We tested if rhamnolipid-mediated tendril formation could be enhanced by artificially stimulating a quorum response on hard agar. Purified 3-oxo-dodecanoyl homoserine lactone and butyryl homoserine lactone (5 µM each) were added to swarm plates prepared at both agar types. This exogenous addition of acyl homoserine lactone (AHL) quorum sensing signals did not promote tendril formation (Figure 4d). While the AHL-deficient *lasIrhlI* mutant strain was restored to wild-type swarming levels, wild-type swarm patterns on either agar type remain generally unchanged with added AHL (Figure 4). Thus, while we cannot rule out some diffusion differences between these soft and hard agar plates, the regulation of quorum sensing and rhamnolipid production on harder agar cannot be explained solely by diffusion as exogenously supplying more AHL showed no effect.

**Figure 5:**
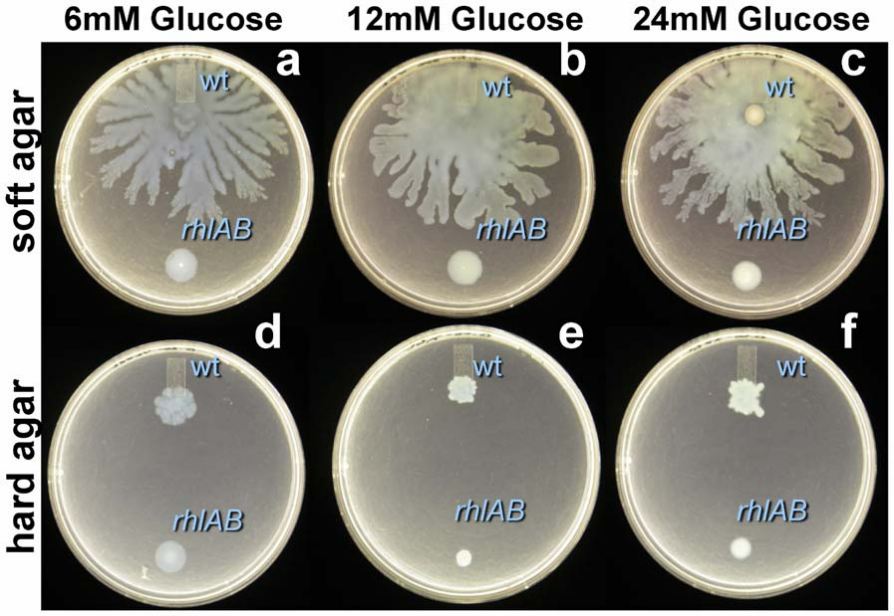
*P. aeruginosa* swarming on FAB with (a, d) 6 mM, (b, e) 12 mM, or (c,f) 24 mM glucose on (a,b,c) soft (0.45%) and (d,e,f) hard (0.6%) agar by wild-type and *rhlAB*-mutant. Plate assays were incubated at 30°C for 60 hours.

We were also unable to induce tendril formation by increasing substrate. We tested if we could increase growth on hard agar by adding more carbon to encourage growth of a sufficient rhamnolipid producing quorum. Variations to the carbon concentration used in swarm plate assays had no apparent impact upon swarming (Figure 5). Neither lowering nor increasing the glucose concentration from 12 mM altered the tendril patterns formed on either agar type.

### Differentiating hard and soft agar

Even with the variations to agar concentration used for these plate assays, the overall nutrient composition is consistent—agar is not metabolized by *P. aeruginosa*. This suggests a physical influence from the altered surface properties of the growth medium that yields the observed tendril patterns. One distinction that can be made for these agar plates is their contact angle, which measures the internal angle of liquid at a liquid-solid interface. The contact angle of a water droplet on top of these plates (with no bacterial inoculum present) was 39.7°±2.8° (avg±SD) and 18.8°±7.3° for soft and hard agar, respectively.

We recreated hard and soft agar conditions for swarming using the agar substitute Gelzan (Sigma). A range of 0.1%–0.2% Gelzan was sufficient to yield both tendril and non-tendril swarms (Figure S5); this showed that tendril swarms can form on semi-solid surfaces other than agar. This alternate swarm assay result also suggests that differences in wettability or hydrophobicity are insufficient to solely explain the observed differences in rhamnolipid-tendril swarming. While the soft agar (or Gelzan) surfaces show a higher contact angle (i.e., more hydrophobicity and less wettability [34]) than for harder surfaces, the contact angle for all Gelzan plates was below 10°. There was not a specific surface contact angle that promoted tendril formation.

## Discussion

*P. aeruginosa* swarm motility is controlled by many factors. Recent reports describe many genetic and environmental components important to swarming [9], [18], [21], [24], [35], [36], [37]. Within these and other studies are various assay conditions reported to promote swarming for *P. aeruginosa*. Indeed, the basic plate assay used to study swarming for various bacteria requires a particular agar concentration range that can depend upon agar purity and media composition [e.g. 11], [19], [20], [38]. The variability of swarming due to specific chemical constituents (e.g., phosphate availability, carbon source, nitrogen source, presence of specific amino acids, and oxygen tension) is becoming well documented [10], [18], [22], [23]. Additionally, water availability is highly influential for swarming; Tremblay et al. [26] documented how drying time prior to inoculation significantly effects the basic swarm assay as over-dried plates do not allow cells to swarm. Similarly, over-wet plates allow for spreading without bacterial surface motility [39]. Here we demonstrate that the formation of swarm tendrils observed with changes to the growth surface is due to localized differences in quorum sensing and subsequent rhamnolipid production on these surfaces.

The formation of tendrils during swarming is primarily dependent upon liquid effects. The production of rhamnolipid lowers the surface tension within the swarm liquid layer [10], [40], [41] and rhamnolipid also likely acts osmotically to increase water content (i.e., volume) of this liquid layer by drawing water from the agar matrix as in [8], similar to the properties of other anionic surfactants [42]. This rhamnolipid-rich, low surface tension, swarm liquid layer then spreads in a classic two-phase tendril pattern described first by Marangoni in the 19^th^ Century [43], [44]. Here we show that fractal formation and increased spreading is dependent on a subpopulation of cells that show robust *rhlA* transcription at the swarm zone edge. Rhamnolipid becomes a dominant factor in motility when cells transcribe *rhlA* (leading to rhamnolipid production) within close proximity (approximately 10 cell lengths or less) of the advancing swarm edge as on soft agar (Figure 2). Our results are largely in agreement with those of Tremblay and Déziel, who showed decreased expression of *rhlA*, *rhlB*, and *rhlC* in proximity of the swarm edge [36]. Our inspection of a *rhlA-gfp* reporter always showed greatest fluorescence at the swarm center but the fluorescence at the swarm edge varied. Specifically, if *rhlA* is transcribed only far away (≥500 µm) from the swarm edge, as seems to occur on harder agar, cells advance more uniformly by other means without forming rhamnolipid-rich tendrils.

The variation in rhamnolipid production at the swarm edge is explained by differences in quorum sensing on these surfaces. This makes sense as *rhlA* and *rhlB* are transcribed only in the presence of an active “quorum.” Regions exhibiting high fluorescence of an early quorum sensing reporter (P*_rsaL_*::*yfp*) matched those areas indicative of high rhamnolipid. The difference in timing for expression patterns of *rsaL* and *rhlA* reporter fluorescence are in agreement with the onset of these early and late quorum sensing genes [31], [32]. Curiously, we observe quorum sensing induction is not simply a population-based event on these semi-solid surfaces. Large populations of cells, seemingly sufficient for a quorum, are present on all surfaces examined (c.f. Figure 3b and 3f). Thus, the mere presence of a threshold quorum sensing population does not yield tendrils and increased swarming on hard agar. The influence of quorum sensing upon swarm motility has previously been shown to be conditional with changes to the carbon source [18]. These results showing decreased fluorescence of transcriptional reporters for *rsaL* and *rhlA* on hard agar suggest a limitation in quorum sensing induction for hard agar-grown populations. Our results for tendril-forming soft agar swarms are largely in agreement with another study that showed upregulation of quorum sensing genes under swarming conditions [35]. It is not yet clear, however, why quorum sensing is limited on harder agar. Quorum sensing limitation during swarming has been used to explore the role of “cheaters” that bring about a “collapse” of swarming when quorum sensing is not sufficient [45]. A potential explanation for our results and others may be surface variable activity of RsmA, a protein shown regulate quorum sensing and rhamnolipid production [46], [47].

Limitations to quorum sensing induction seemed not to be explained by limited exposure of bacteria to AHL signals on hard agar. Based on a study by Dulla and Lindow [48] that investigated *P. syringae* aggregation upon plant leaves, one might expect quorum sensing to initiate more quickly with limited diffusion and limited surface water. Here we observe the opposite: cell aggregates that develop on hard agar show limited quorum sensing induction. Further, the addition of exogenous AHL does not lead to increased tendril swarming. This attempt to artificially induce quorum sensing with exogenous signal for cells growing on hard agar plates did not promote tendril formation (but it was sufficient to rescue a *lasIrhlI* mutant to a wild-type phenotype). Lastly, rhamnolipid tendril swarms were not affected with changes to the carbon substrate concentration, and conversely, these changes did not stimulate tendril formation on hard agar.

There is clearly a need to understand the development of sub-populations that influence swarming. Additionally, the recent report by Glick et al. [49] of the importance of rhamnolipid to type IV pili-mediated twitching motility may point to a more general response of *P. aeruginosa* to increase rhamnolipid and surface motility under conditions of micronutrient limitations (such as iron) that were not examined here. We propose that rhamnolipid production requires more than just a minimum threshold quorum followed by some adequate interval of time. Providing more time or nutrients for cell growth or exogenous AHL signal does not promote rhamnolipid tendril formation or better swarming. These results suggest that the regulation of rhamnolipid production has specificity to the physical surface characteristics sensed by swarming *P. aeruginosa*. Even when considering the environmental factors and genes known to regulate quorum sensing, rhamnolipid production, and swarming, the true governance of *P. aeruginosa* swarming remains complex and only partially understood.

## Materials and Methods

### Bacterial Strains

*Pseudomonas aeruginosa* ATCC strain 15692 and isogenic mutants or chromosomal reporters for this wild-type background were used for all experiments. All strains and plasmids used are included as Table 2.

**Table 2:** Strain List.

Bacterial Strain	Relevant characteristics	Source or reference
*P. aeruginosa*		
wild-type	ATCC 15692 wild-type 1C	[18]
*lasIrhlI*	15692 *ΔlasI ΔrhlI*; Gm^r^, Tc^r^	[18]
*lasRrhlR*	15692 *ΔlasR ΔrhlR*; Gm^r^, Tc^r^	[18]
wt-gfp	15692 miniTn7 *gfp2*; Cm^r^, Gm^r^	[18]
*rhlAB*	15692 *ΔrhlAB*; Gm^r^	[18]
wt-P*rhlA*-gfp	15692 P*_rhlA_-gfp* reporter; Tc^r^	This study
wt-P*rsaL*-gfp	15692 P*_rsaL_-yfp*(AAV) reporter; Tc^r^	This study
*E. coli*		
SM10	λpir donor strain	[55]
DH5α	cloning host	Invitrogen

Plasmids		
prhlA-gfp	miniCTX-P*_rhlA_*::*gfp*; Tc^r^	[29]
pmini-CTX1	ΦCTX chromosomal integration vector; Tc^r^	[51]
pKDT17	3-oxo-C12 AHL bioassay indicator	[52], [53]
pGJB5	expression plasmid *rsaL* promoter	Balzer and Parsek
pMH487	promoterless GFP expression vector	Hentzer
pJB1197	YFP(ASV) containing plasmid	Anderson
pJDS1	pMH487 with *yfp*(ASV); Ap^r^, Cm^r^, Gm^r^	This study
pJDS4	pJDS1 with P*_rsaL_*::*yfp*(ASV); Ap^r^, Cm^r^, Gm^r^	This study
pJDS5	miniCTX-P*_rsaL_*::*yfp*(ASV); Tc^r^	This study

### Genetic Techniques

Standard genetic techniques were used to construct a *rsaL*-promoter short-half life reporter strain to be used as an indicator of early quorum sensing activity. The promoterless short-half life Yfp construct (pJDS1) was constructed by ligating the sticky-end *yfp*(ASV) SphI & HindIII fragment of JB1193 (similar to pJBA113 in [50]) into pMH487. A 146 bp EcoRI-XbaI fragment from pGJB5 containing the promoter region for *rsaL* was cloned into pJDS1 to create pJDS4. The EcoRI-HindIII fragment containing P*_rsaL_*::*yfp*(ASV) from pJDS4 was cloned into the MCS of pmini-CTX1 to create pJDS5.

The resultant chromosomal fluorescent reporters for P*_rhlA_* and P*_rsaL_* expression were made by integrating the plasmids prhlA-gfp [29] and pJDS5, respectively, into *P. aeruginosa* by conjugational mating and selection of tetracycline resistant colonies [51].

### Swarming Assays

Swarming was studied using plate assays containing 0.4%–0.6% noble agar and FAB medium with 12 mM glutamate or glucose [18]. Plates were inoculated using a sterilized platinum wire with log-phase cells (OD_600_≈0.7) grown in FAB medium containing the respective carbon source (30 mM) approximately 8 hours after pouring the plates and incubated at 30°C. All experiments were performed in a minimum of triplicate.

### Microscopy

Images of cells containing fluorescent reporter constructs were acquired with a Nikon 80i microscope equipped with 10× or 40× objective and were recorded with a CoolSnap HQ camera (Photometrics) operated with Metamorph software. The swarm zone edge or wet mounts of batch cultures were imaged first in phase contrast and then fluorescence (of both Gfp and Yfp reporters) using a ET-EYFP (Chroma # 49003) filter set. Representative images were minimally processed; all optimum settings (e.g. brightness, contrast) were determined empirically to capture the maximum fluorescence range for each set of experiments and uniformly applied to all images to accurately represent experimental results.

### Rhamnolipid imaging

Methylene blue-containing rhamnolipid indicator plates were prepared by adding cetyltrimethylammonium bromide (CTAB) and methylene blue (Sigma) as described previously [27] to 0.4% and 0.6% Noble agar FAB-glutamate plates.

### Contact Angle Measurements

Contact angle measurements were obtained using a goniometer (FTÅ200, First Ten Ångstroms). A syringe pump was used to dispense a consistent droplet of water, which was deposited onto the swarm plate surfaces and the resultant contact angle was measured after 10 seconds. Five droplet measurements were recorded for 0.4% agar and 0.6% agar.

### Fluorescence, cell density, and luminescence measurements

Flourescence (excitation = 485 nm, emission = 535 nm), optical density (590 nm), and luminescence were recorded using a TECAN GENios Pro plate reader. At the time points indicated, aliquots of batch cultures were distributed into 96-well, black plastic, clear bottom plates. Concentrations of 3-oxo-dodecanoyl (3-oxo-C12) homoserine lactone were determined by calibrating against dilutions of a known concentration standard using luminescence of the pKDT17 reporter bioassay [52], [53]. The P*_rsaL_*::*yfp*(ASV) reporter is extremely sensitive to 3-oxo-C12; fluorescence is apparent for planktonic cells grown in batch cultures below 150 nM 3-oxo-C12 signal (Figure S6).

### Fluorescence Image Analysis

Data from fluorescence images of the *rhlA* and *rsaL* reporters were quantified using ImageJ [54]. Raw TIFF files were opened in ImageJ and the background fluorescence intensity was determined for all the images collected for each separate reporter. The histogram from each image was exported to a spreadsheet and overall fluorescence intensity was integrated (bin range×number of pixels) for pixels above the background threshold for each image.

## Supporting Information

Movie S1 **Motility of** ***P. aeruginosa*** **wild-type growing on soft (0.4%) agar with FAB-glutamate at the swarm edge after 40 hours incubation.** Phase images were recorded at 15 frames per second for 20 seconds.
(AVI)

Movie S2 **Motility of** ***P. aeruginosa*** **wild-type growing on hard (0.6%) agar with FAB-glutamate at the swarm edge after 40 hours incubation.** Phase images were recorded at 15 frames per second for 20 seconds.
(AVI)

Figure S1 ***P. aeruginosa*** **swarming for wild-type,** ***rhlAB*** **-mutant,** ***fliM*** **-mutant, and** ***fliM*** **+** ***rhlAB*** **-double mutant on soft agar (0.40%) and hard agar (0.60%) with FAB-glucose medium.** Plate assays were incubated at 30°C for 36 hours.
(PDF)

Figure S2 **Methylene blue-rhamnolipid plate assay at soft (0.4%) and hard (0.6%) agar for wild-type,** ***lasIrhlI*** **-mutant, and** ***rhlAB*** **-mutant strains.** The presence of surfactant is indicated by a ring of clearing.
(PDF)

Figure S3 **GFP expressionnear the swarm center for a rhamnolipid fluorescence reporter during** ***P. aeruginosa*** **wild-type strain swarming growing on soft (0.4%) and hard (0.55%) agar.** (a,d) fluorescence of P*_rhlA_*::*gfp* fusion;(b,e) phase-contrast image; (c,g) overlay of a+band d+e.
(PDF)

Figure S4 ***P. aeruginosa*** **swarming after 12, 27, and 63 hours for a rhamnolipid fluorescence reporter wild-type strain growing on soft (0.4%) and hard (0.6%) agar.** Lettered panels show the entire swarm colony; (i) phase-contrast image of swarm edge; (ii) fluorescence of P*_rhlA_*::*gfp* fusion; (iii) overlay of i+ii panels for each image showing proximity of rhamnolipid production to the swarm edge. Overlay scale bar = 200 µm.
(PDF)

Figure S5 ***P. aeruginosa*** **swarming for wild-type and** ***rhlAB*** **-mutant on different % Gelzan with FAB-glucose medium.** Plate assays were incubated at 30°C for 48 hours.
(PDF)

Figure S6 **Expression of the quorum sensing transcriptional reporter P*_rsaL_*::*yfp*. Planktonic bacterial cultures were grown in FAB-glutamate medium in shaker flasks at 30°C.** Increases in optical density of the bacterial culture and fluorescence intensity of the reporter are shown over time for the wild-type and quorum sensing (*lasRrhlR*) mutant strains harboring the chromosomal insertion. Additional boxes show the fluorescence and phase images of wet mounts prepared from these cultures at the times indicated; the 3-oxo-C12 homoserine lactone concentration detected by bioassay at these time points is also indicated.
(PDF)
