# Supplementary material for: Surface Hardness Impairment of Quorum Sensing and Swarming for Pseudomonas aeruginosa
Source: PLoS One. 2011 Jun 7;6(6):e20888. doi: 10.1371/journal.pone.0020888 (PMC3110244; doi:10.1371/journal.pone.0020888)
Supplement: Figure S1 — P. aeruginosa swarming for wild-type, rhlAB -mutant, fliM -mutant, and fliM + rhlAB -double mutant on soft agar (0.40%) and hard agar (0.60%) with FAB-glucose medium. Plate assays were incubated at 30°C for 36 hours. (PDF) [file pone.0020888.s003.pdf]

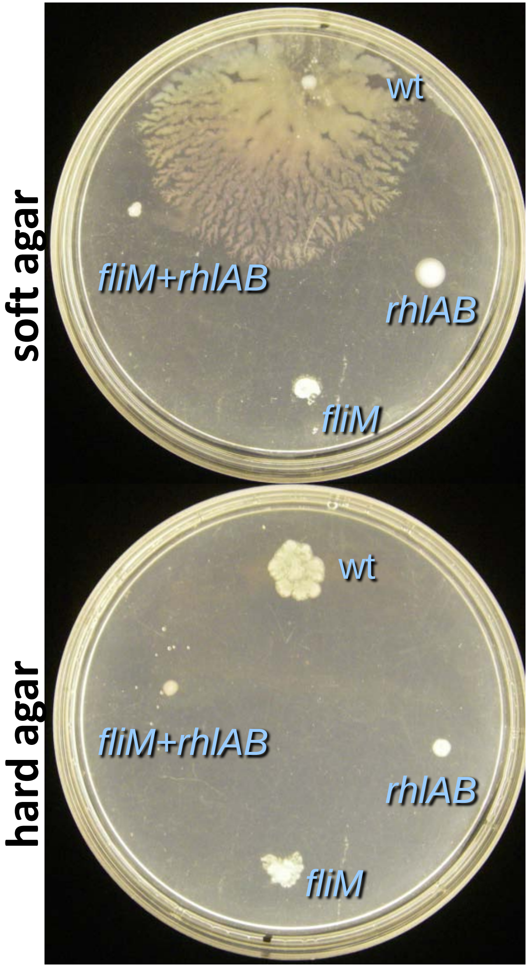


**Figure S1.** ***P. aeruginosa*** **swarming for wild-type, *rhlAB*-mutant, *fliM*-mutant, and *fliM+rhlAB*-double mutant on soft agar (0.40%) and hard agar (0.60%) with FAB-glucose medium. Plate assays were incubated at 30°C for 36 hours.**
