# Supplementary material for: Surface Hardness Impairment of Quorum Sensing and Swarming for Pseudomonas aeruginosa
Source: PLoS One. 2011 Jun 7;6(6):e20888. doi: 10.1371/journal.pone.0020888 (PMC3110244; doi:10.1371/journal.pone.0020888)
Supplement: Figure S2 — Methylene blue-rhamnolipid plate assay at soft (0.4%) and hard (0.6%) agar for wild-type, lasIrhlI -mutant, and rhlAB -mutant strains. The presence of surfactant is indicated by a ring of clearing. (PDF) [file pone.0020888.s004.pdf]

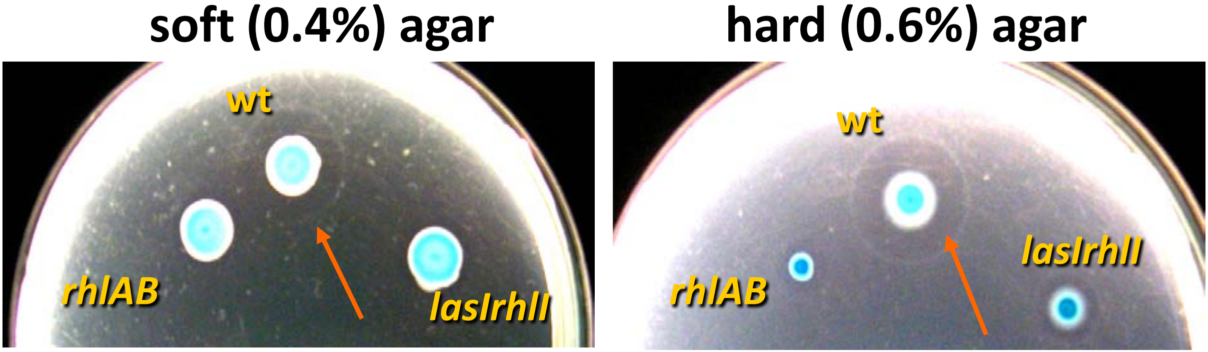

Figure S2. Methylene blue-rhamnolipid plate assay at soft (0.4%) and hard (0.6%) agar for wild-type, *lasIrhlI*-mutant, and *rhlAB*-mutant strains. The presence of surfactant is indicated by a ring of clearing (marked by the arrow).
