# Supplementary material for: Surface Hardness Impairment of Quorum Sensing and Swarming for Pseudomonas aeruginosa
Source: PLoS One. 2011 Jun 7;6(6):e20888. doi: 10.1371/journal.pone.0020888 (PMC3110244; doi:10.1371/journal.pone.0020888)
Supplement: Figure S3 — GFP expressionnear the swarm center for a rhamnolipid fluorescence reporter during P. aeruginosa wild-type strain swarming growing on soft (0.4%) and hard (0.55%) agar. (a,d) fluorescence of PrhlA::gfp fusion;(b,e) phase-contrast image; (c,g) overlay of a+band d+e. (PDF) [file pone.0020888.s005.pdf]

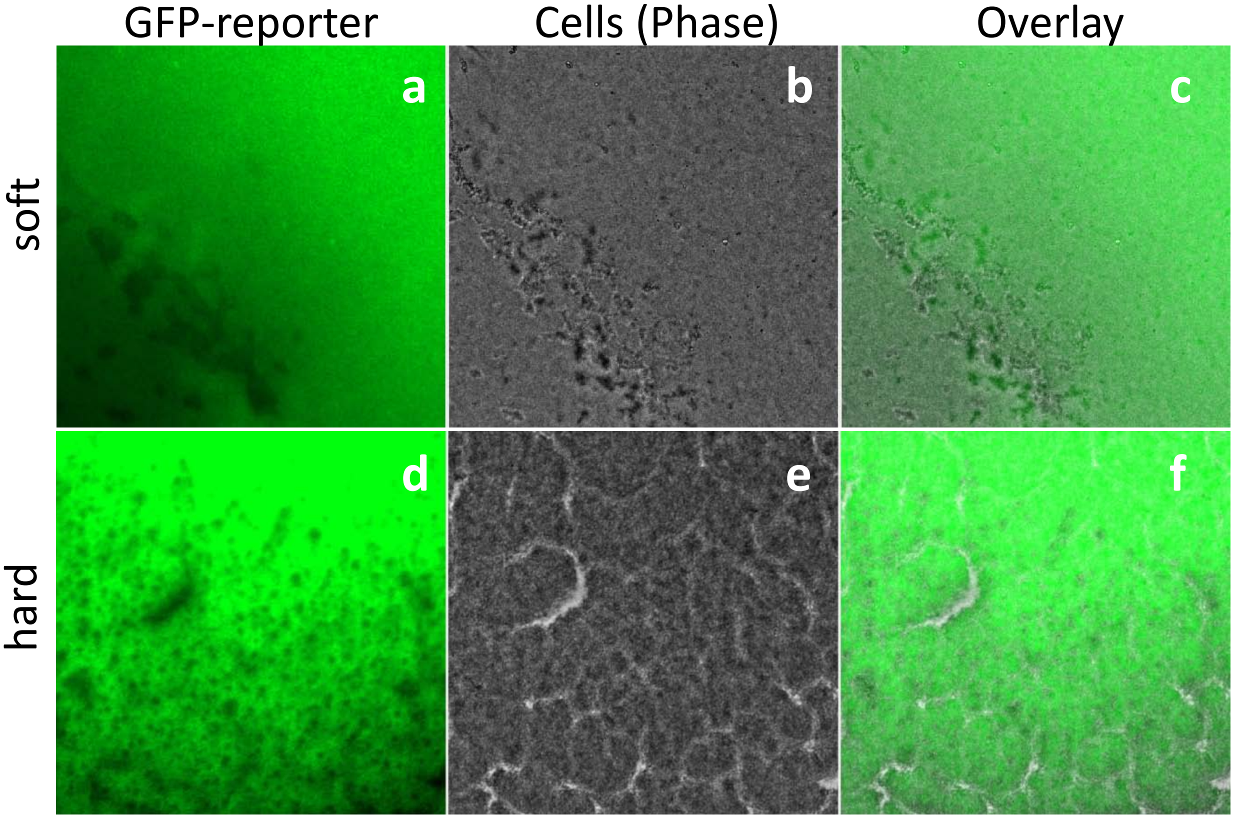

Figure S3. GFP expression near the swarm center for a rhamnolipid fluorescence reporter during *P. aeruginosa* wild-type strain swarming growing on soft (0.4%) and hard (0.55%) agar. (a,d) fluorescence of P_{rhlA}::*gfp* fusion; (b,e) phase-contrast image; (c,g) overlay of a+b and d+e.
