# Supplementary material for: Surface Hardness Impairment of Quorum Sensing and Swarming for Pseudomonas aeruginosa
Source: PLoS One. 2011 Jun 7;6(6):e20888. doi: 10.1371/journal.pone.0020888 (PMC3110244; doi:10.1371/journal.pone.0020888)
Supplement: Figure S4 — P. aeruginosa swarming after 12, 27, and 63 hours for a rhamnolipid fluorescence reporter wild-type strain growing on soft (0.4%) and hard (0.6%) agar. Lettered panels show the entire swarm colony; (i) phase-contrast image of swarm edge; (ii) fluorescence of PrhlA::gfp fusion; (iii) overlay of i+ii panels for each image showing proximity of rhamnolipid production to the swarm edge. Overlay scale bar = 200 µm. (PDF) [file pone.0020888.s006.pdf]

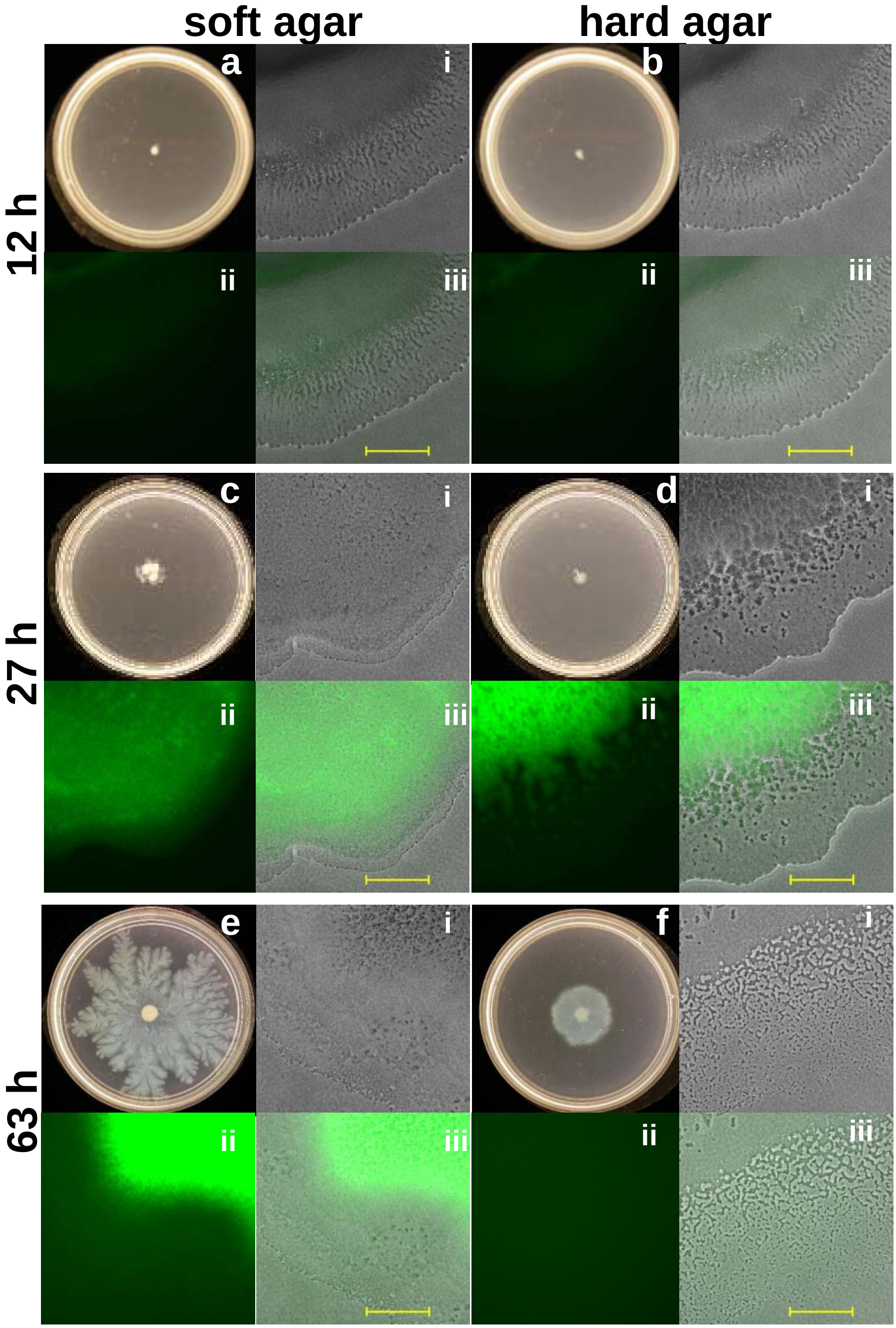

Figure S4. ***P. aeruginosa*** **swarming after 12, 27, and 63 hours for a rhamnolipid fluorescence reporter wild-type strain growing on soft (0.4%) and hard (0.6%) agar. Lettered panels show the entire swarm colony; (i) phase-contrast image of swarm edge; (ii) fluorescence of P_{rhlA}::*gfp* fusion; (iii) overlay of i+ii panels for each image showing proximity of rhamnolipid production to the swarm edge. Overlay scale bar = 200 µm.**
