# Supplementary material for: Surface Hardness Impairment of Quorum Sensing and Swarming for Pseudomonas aeruginosa
Source: PLoS One. 2011 Jun 7;6(6):e20888. doi: 10.1371/journal.pone.0020888 (PMC3110244; doi:10.1371/journal.pone.0020888)
Supplement: Figure S5 — P. aeruginosa swarming for wild-type and rhlAB -mutant on different % Gelzan with FAB-glucose medium. Plate assays were incubated at 30°C for 48 hours. (PDF) [file pone.0020888.s007.pdf]

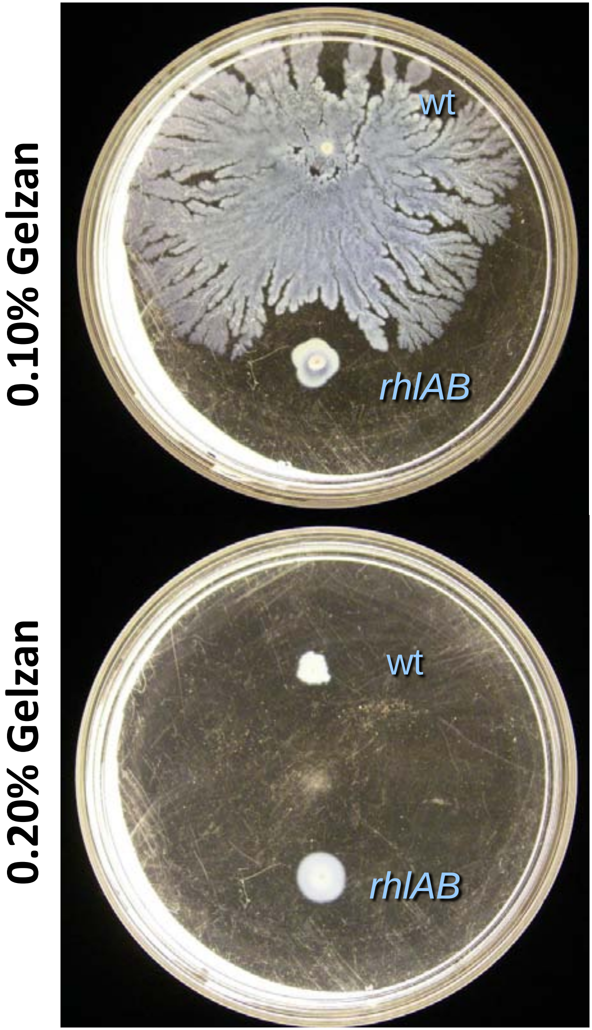


**Figure S5.** *P. aeruginosa* swarming for wild-type and *rhlAB*-mutant on different % Gelzan with FAB-glucose medium. Plate assays were incubated at $30^{\circ}C$ for 48 hours.
