# Supplementary material for: Surface Hardness Impairment of Quorum Sensing and Swarming for Pseudomonas aeruginosa
Source: PLoS One. 2011 Jun 7;6(6):e20888. doi: 10.1371/journal.pone.0020888 (PMC3110244; doi:10.1371/journal.pone.0020888)
Supplement: Figure S6 — Expression of the quorum sensing transcriptional reporter PrsaL::yfp. Planktonic bacterial cultures were grown in FAB-glutamate medium in shaker flasks at 30°C. Increases in optical density of the bacterial culture and fluorescence intensity of the reporter are shown over time for the wild-type and quorum sensing (lasRrhlR) mutant strains harboring the chromosomal insertion. Additional boxes show the fluorescence and phase images of wet mounts prepared from these cultures at the times indicated; the 3-oxo-C12 homoserine lactone concentration detected by bioassay at these time points is also indicated. (PDF) [file pone.0020888.s008.pdf]

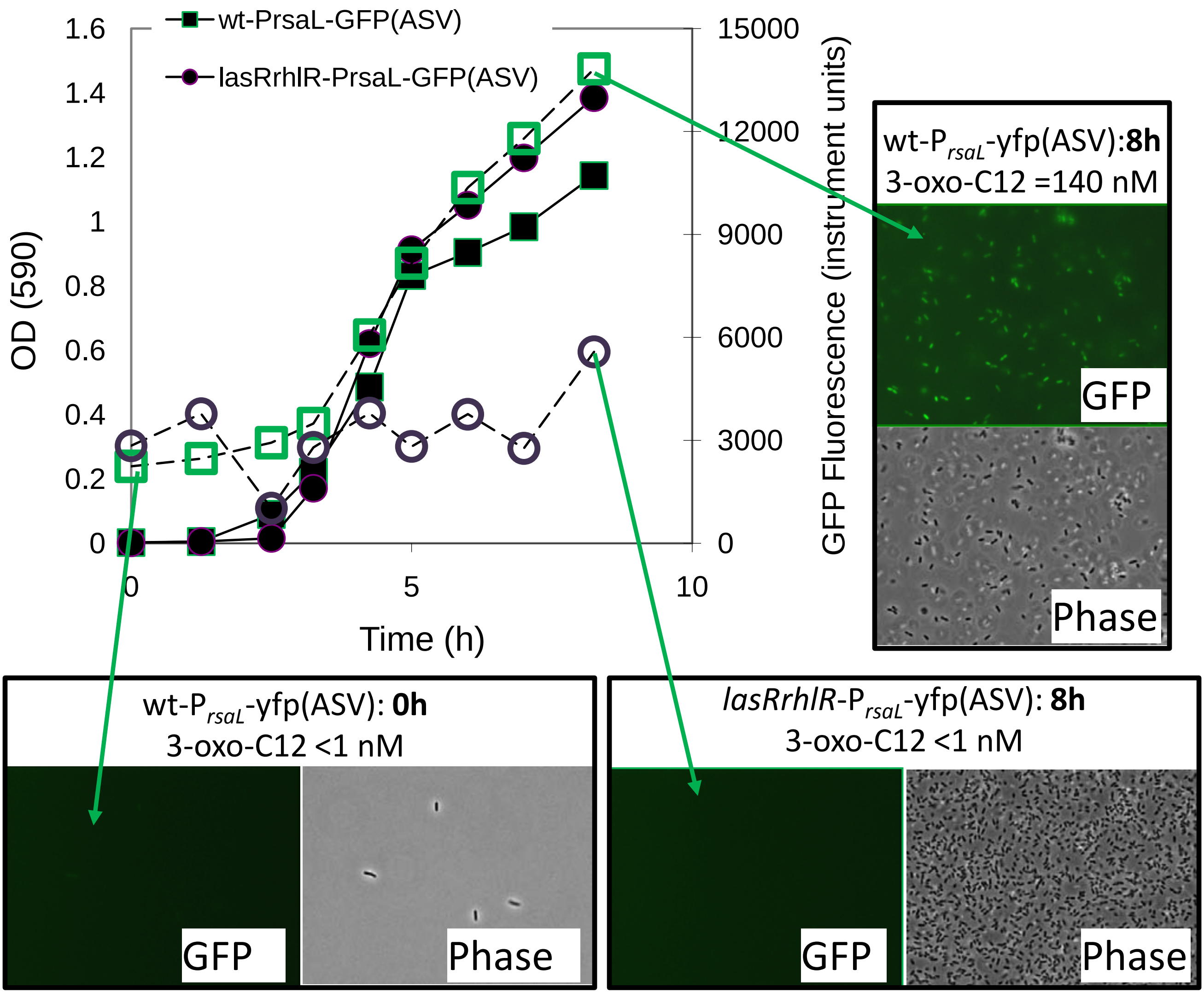

Figure S6. Expression of the quorum sensing transcriptional reporter P$_{rsaL}$::*yfp*. Planktonic bacterial cultures were grown in FAB-glutamate medium in shaker flasks at 30°C. Increases in optical density of the bacterial culture and fluorescence intensity of the reporter are shown over time for the wild-type and quorum sensing (*lasRrhlR*) mutant strains harboring the chromosomal insertion. Additional boxes show the fluorescence and phase images of wet mounts prepared from these cultures at the times indicated; the 3-oxo-C12 homoserine lactone concentration detected by bioassay at these time points is also indicated.
